# Prevalence of *Helicobacter pylori* Infection in Geriatric Adults: A Single-Center Cohort Study from Türkiye

**DOI:** 10.5152/tjg.2025.25499

**Published:** 2026-01-05

**Authors:** Yavuz Özden, Oktay Bulur

**Affiliations:** 1 Department of Gastroenterology, University of Health Sciences Kayseri Faculty of Medicine, Kayseri City Training and Research Hospital, Kayseri, Türkiye; 2 Department of Gastroenterology, İstanbul Medipol University, İstanbul, Türkiye

**Keywords:** Endoscopy, *Helicobacter pylori*, histopathology, older adults, prevalence, Türkiye

## Abstract

**Background/Aims:**

*Helicobacter pylori* infection remains an important global health issue, particularly among older adults for whom related complications are more frequent. However, epidemiological data on the elderly populations in Türkiye are limited. This study aimed to determine the prevalence, associated factors, and endoscopic–histopathological correlations of *H*.* pylori* infection in a large geriatric cohort.

**Materials and Methods:**

This single-center retrospective study included 2000 adults aged ≥65 years who underwent esophagogastroduodenoscopy between January 2023 and December 2024. Active infection was determined by stool antigen testing according to the Updated Sydney System. Demographic, clinical, endoscopic, and histopathological parameters were analyzed, and independent predictors were identified using multivariate logistic regression.

**Results:**

The mean age of the participants was 77.5 ± 7.8 years, and 50.2% were male. The overall prevalence of *H*.* pylori* infections was 56.3%. Prior eradication therapy was independently protective (31.3% vs. 66.1%; *P* < .001). Infection rates did not differ significantly according to sex, smoking, alcohol use, or comorbidities. The highest positivity rates were observed for atrophic gastritis (60.8%), pangastritis (58.0%), and antral gastritis (56.2%). The prevalence declined modestly in the oldest-old group (≥85 years), whereas atrophic and metaplastic changes increased with age.

**Conclusion:**

*Helicobacter pylori* infection remains highly prevalent among elderly individuals in Türkiye, showing a gradual decline compared with historical national data but a persistent association with premalignant mucosal changes. Early detection and individualized eradication strategies are essential to optimize geriatric gastrointestinal care.

**Main Points**

- *Helicobacter pylori* infection remains highly prevalent among elderly individuals in Türkiye, with an overall rate of 56.3%.
- The infection prevalence showed a modest decline in the oldest-old group (≥85 years), whereas atrophic and metaplastic mucosal changes increased with age.
- Prior eradication therapy significantly reduced the infection rates, confirming its long-term protective effects.
- Traditional demographic and lifestyle factors such as sex, smoking, alcohol use, and polypharmacy were not independent predictors of infection.
- Integration of systematic diagnostic testing and age-appropriate eradication strategies is essential to improve geriatric gastrointestinal care.

## Introduction

*Helicobacter pylori* (*H*.* pylori*) is a gram-negative bacterium that causes chronic gastritis, peptic ulcer disease, and gastric malignancy worldwide.^1^^,^^2^ Infection is commonly acquired in childhood and may persist for decades if untreated. Its clinical consequences, including peptic ulcer bleeding and gastric cancer, are particularly pronounced among older adults, in whom age-related mucosal atrophy and comorbidities contribute to increased vulnerability.^3^^,^^4^

The prevalence of *H*.* pylori* varies geographically and is influenced by sanitation, socioeconomic status, and antibiotic exposure.^5^ In Türkiye, historical studies reported infection rates above 80%, but contemporary data reveal a progressive decline to approximately 50%-60%.^6^^-10^ Özaydin et al^6^ found a national prevalence of 82.5%, while Özden et al^7^ documented a significant serological decrease over a decade. Erkut et al^8^ reported rates of approximately 40% in symptomatic adults, and Çubuk et al^9^ confirmed a similar reduction in recent years. Nevertheless, Türkiye remains within the high-prevalence category according to the Maastricht VI/Florence Consensus, with antibiotic resistance posing an ongoing challenge.^11^

Despite substantial research on *H*.* pylori*, studies focusing specifically on the geriatric populations are scarce. Most evidence arises from younger or mixed cohorts, leaving the age-related distribution of infections, endoscopic patterns, and histopathological correlates that have not been sufficiently explored. This study aimed to determine the prevalence, risk factors, and clinicopathological associations of *H*.* pylori* infection in a large cohort of Turkish adults aged ≥65 years.

## Materials and Methods

### Study Design and Population

This single-center retrospective cohort study was conducted at the University of Health Sciences, Kayseri City Hospital, Department of Gastroenterology between January 2023 and December 2024. Patients aged ≥65 years who underwent esophagogastroduodenoscopy (EGD) for upper gastrointestinal (GI) symptoms or clinical indications, including selected screening cases, were evaluated for eligibility. A total of 2000 patients met the inclusion criteria and were analyzed.

Exclusion criteria were as follows: (1) active upper GI bleeding at presentation; (2) upper GI surgery within the past month; (3) cardiopulmonary instability precluding endoscopy; (4) use of antibiotics, bismuth compounds, or proton pump inhibitors (PPIs) within 4 weeks prior to evaluation; and (5) incomplete demographic or histopathological data.

A history of *H*.* pylori* eradication was not an exclusion criterion and was recorded as a variable in the analysis.

Ethical approval was obtained from the Institutional Review Board of the University of Health Sciences, Kayseri City Hospital (Approval no. 2024/216; December 24, 2024). Written informed consent was obtained from all participants at the time of the endoscopic procedures as part of the routine hospital documentation. Since this was a retrospective analysis of anonymized data, the Ethics Committee waived the need for additional informed consent. All the procedures complied with the principles of the Declaration of Helsinki.

### Data Collection

Demographic variables (age and sex), clinical indications, and comorbidities were retrieved from standardized electronic records. Symptoms were classified as dyspepsia, reflux, abdominal pain, nausea, diarrhea, or asymptomatic. The comorbidities included hypertension, diabetes mellitus, chronic kidney disease, and chronic obstructive pulmonary disease.

Multimorbidity was defined as ≥2 chronic conditions, and polypharmacy as the concurrent use of ≥5 medications. Lifestyle factors (smoking, alcohol use) and history of *H*.* pylori* eradication therapy were also documented.

### Endoscopic and Histopathological Assessment

All procedures were performed by board-certified gastroenterologists with at least 5 years of endoscopy experience, and a minimum of 1000 diagnostic EGD procedures were performed.

Endoscopic descriptions such as “antral gastritis,” “corpus gastritis,” and “atrophic appearance” were used as macroscopic descriptors rather than definitive diagnoses. The histological confirmation of inflammation was subsequently established using the Updated Sydney Classification.^12^

The histopathological evaluation was performed by a single GI pathologist who was blinded to the clinical data. The features evaluated included chronic and active inflammation, glandular atrophy, intestinal metaplasia, and dysplasia. For *H*.* pylori* detection, Giemsa staining was routinely used, and visualization of bacilli was accepted as evidence of infection.^13^

### *Helicobacter pylori* Diagnosis

Because elderly patients frequently use acid-suppressive drugs that can reduce biopsy sensitivity, stool antigen testing was used to complement histology.^5^ All patients provided stool samples before endoscopy. Testing was performed using a rapid immunochromatographic assay (sensitivity >90%, specificity >95%), unaffected by concurrent PPI use.

*Helicobacter pylori* positivity was defined as follows:

1. A positive stool antigen result, or
2. Histological identification of bacilli or marked active gastritis in the presence of a negative stool test result, or
3. Discordant results were reviewed jointly by an endoscopist and pathologist to reach a consensus diagnosis.

### Statistical Analysis

Data were analyzed using IBM SPSS Statistics, version 25.0 (IBM SPSS Corp.; Armonk, NY, USA). Continuous variables are presented as mean ± standard deviation for normally distributed data and as median (interquartile range, IQR) for non-normal data. Categorical variables are expressed as frequencies and percentages.

Comparisons between groups were made using Student’s *t*-test or Mann–Whitney *U-*test for continuous variables and the *χ*^2^ test for categorical variables.

Multivariate logistic regression analysis identified the independent predictors of *H*.* pylori* positivity. The candidate variables included age, sex, smoking, alcohol use, polypharmacy, comorbidities, and prior eradication therapy. Odds ratios with 95% CI are reported. Model calibration was tested using the Hosmer–Lemeshow test, and discrimination was evaluated using the area under the receiver operating characteristic curve.

All statistical tests were 2-sided, with *P* < .05 considered significant. Subgroup analyses were pre-specified for age (65-74, 75-84, and ≥85 years) and polypharmacy status (≥5 vs. <5 medications). Trend analyses across age groups were conducted using the Cochran–Armitage test.

## Results

A total of 2000 elderly patients were included in the analysis (mean age, 77.5 ± 7.8 years; range, 65-90 years). Of these, 1004 (50.2%) were male and 996 (49.8%) were female. The most frequent symptoms were dyspepsia (25.1%) and reflux (20.0%), followed by abdominal pain (15.8%), diarrhea (15.6%), and nausea (13.4%). A total of 202 patients (10.1%) were asymptomatic and underwent endoscopy for screening.

Multimorbidity (≥2 chronic diseases) was present in 514 patients (25.7%), with hypertension (20.9%), diabetes mellitus (14.5%), chronic obstructive pulmonary disease (9.8%), and chronic kidney disease (4.2%) being the most frequent comorbidities. Polypharmacy (≥5 drugs) was observed in 812 (40.6%) patients. A total of 562 patients (28.1%) reported a history of *H*.* pylori* eradication therapy, 610 (30.5%) were current smokers, and 298 (14.9%) reported regular alcohol use. The baseline characteristics are summarized in Table 1.

The patients were stratified into 3 age groups: 65-74 years (n = 800), 75-84 years (n = 800), and ≥85 years (n = 400). The age-stratified baseline features are presented in Table 2.

### Prevalence of *Helicobacter pylori*

The overall prevalence of *H*.* pylori* infection was 56.3% (n = 1126). Prevalence did not differ significantly between males (57.9%) and females (54.6%) (*P* = .18), or between mean ages (77.3 vs. 77.9 years, *P* = .42). Across age groups, the infection rates were 58.2%, 56.0%, and 53.9% for 65-74, 75-84, and ≥85 years, respectively, showing a modest decline with advancing age (*P*-trend = .04; Table 3).

### Association with Clinical Subgroups

Previous eradication therapy was significantly associated with a lower infection rate (31.3% vs. 66.1%; *P* < .001). Smoking, alcohol use, and polypharmacy were not found to be significant predictors of infection. The prevalence was 51.5% in smokers vs. 58.4% in non-smokers (*P* = .09), 53.0% in alcohol users vs. 56.9% in non-users (*P* = .27), and 59.1% in patients with polypharmacy vs. 54.4% in those without polypharmacy (*P* = .11). The details are presented in Table 4.

### Endoscopic Findings

Among all the patients, 66 (3.3%) had normal endoscopic findings, with an *H*.* pylori* positivity rate of 42.4%. The most common abnormal findings were pangastritis (22.4%, positivity = 58.0%), antral changes (20.1%, 56.2%), corpus changes (9.7%, 55.7%), erythematous changes (7.7%, 53.2%), and an atrophy-like appearance (9.7%, 60.8%). Other lesions included reflux esophagitis (7.3%, 39.7%), gastric ulcers (6.0%, 45.0%), duodenal ulcers (4.4%, 54.5%), gastric polyps (5.1%, 39.2%), and duodenitis (4.3%, 51.2%). The findings are summarized in Table 5.

When endoscopic features were compared between *H*.* pylori*-positive and -negative groups, statistically significant differences were observed for atrophic appearance (*P* = .01) and pangastritis (*P* = .04), while other findings were not significantly different (all *P* > .05).

In age-stratified analyses, the frequency of pangastritis and atrophic gastritis increased with age (*P*-trend <.01 and <.001, respectively), whereas reflux esophagitis was more frequent in the younger elderly (10.5% → 5.7% → 4.0%; *P* < .001). The incidence of antral gastritis (22.7% → 20.0% → 15.0%; *P* = .03) and erythematous gastritis (9.0% → 7.7% → 5.0%; *P* = .04) decreased with age. Corpus gastritis, peptic ulcers, gastric polyps, and duodenitis did not differ significantly between the groups (all *P* > .05; Table 6).

### Histopathological Findings

Histopathological evaluation revealed chronic inflammation (35.3%), atrophic gastritis (20.5%), intestinal metaplasia (19.2%), and dysplasia (4.2%). The corresponding *H*.* pylori* positivity rates were 57.0%, 60.5%, 50.0%, and 40.5%, respectively (Supplementary Table 1).

Age-stratified histological analysis showed progressive increases in atrophic gastritis (16.5% → 22.2% → 25.0%; *P* < .001) and intestinal metaplasia (16.0% → 20.5% → 23.0%; *P* < .001) with advancing age, whereas chronic inflammation decreased (39.0% → 35.2% → 28.0%; *P* < .01). Dysplasia rates remained stable across age groups (4.0% → 4.2% → 4.5%; *P* = .77) (Supplementary Table 2).

## Discussion

In this large single-center cohort of 2000 elderly patients, the overall prevalence of *H*.* pylori* infection was 56.3%, which aligns with recent findings in older populations.^3^^,^^4^Historically, infection rates have exceeded 80% in Türkiye, but a progressive decline has been documented over the past 2 decades, paralleling global trends.^1^^,^^2^ This reduction likely reflects improved hygiene, living standards, and the widespread implementation of eradication programs. The modest decrease observed among the oldest-old subgroup (≥85 years) may be attributed to advanced mucosal atrophy or prior antibiotic exposure, which reduces bacterial colonization.^6^^,^^7^

In Türkiye, epidemiological data demonstrate a clear downward trend. Özaydin et al^6^ reported a national prevalence of 82.5% in adults, whereas Özden et al^7^ documented a marked decline in seropositivity over a 10-year period. Erkut et al^8^ and Çubuk et al^9^ later confirmed lower rates (40%-55%) in both community and hospital settings. Nevertheless, *H*.* pylori* infection remains a significant public health issue, as emphasized by Kaplan et al,^10^ who also highlighted the increasing antibiotic resistance. Collectively, these findings reveal a consistent national decline from >80% to approximately 50%-60%, consistent with the prevalence in the geriatric cohort.

Consistent with the previous literature, infection was not significantly associated with sex, smoking, alcohol use, or polypharmacy.^3^^,^^4^ This indicates that traditional demographic and lifestyle factors are poor predictors in older adults, supporting systematic screening rather than selective screening. Prior eradication therapy was strongly protective, with an infection rate of 31% compared with 66% in untreated individuals, reaffirming the long-term efficacy of eradication therapy.

The subgroup analyses revealed that although the overall prevalence declined slightly with age, premalignant histopathologic features, particularly atrophy and intestinal metaplasia, increased. This paradox underscores the importance of early diagnosis and eradication before the development of irreversible mucosal changes.^12^ Endoscopically, *H*.* pylori* was highly prevalent in pangastritis, antral gastritis, and atrophic gastritis, but was also detected in macroscopically normal mucosa. Hence, reliance solely on endoscopic appearance risks underdiagnosis and reinforces the value of combined endoscopic and histological assessment.

It is essential to emphasize that the endoscopic labels “gastritis” or “atrophic appearance” in this study represented macroscopic descriptors rather than definitive pathological diagnoses. Histological confirmation remains the diagnostic standard, in accordance with the Updated Sydney System.^12^

Among elderly patients with peptic ulcer disease, *H*.* pylori* positivity was observed in 55% of duodenal ulcers and 45% of gastric ulcers, which is substantially lower than the >90% typically observed in younger cohorts. These findings support previous evidence suggesting that an increasing proportion of ulcers in the elderly are *H*.* pylori*-negative and likely nonsteroidal anti-inflammatory drugs (NSAID)-related.^13^ Because the coexistence of *H*.* pylori* infection and long-term NSAID use markedly increases ulcer risk, current guidelines recommend eradication therapy for all elderly patients requiring chronic NSAID treatment.^5^

Management of *H*.* pylori* infection in older adults presents unique challenges, including multimorbidity, polypharmacy, frailty, and antibiotic resistance.^14^^-^^16^ Individualized regimens that balance efficacy and safety are essential. Although antibiotic resistance profiling was not available in this study, the significantly lower infection rates among previously eradicated patients highlight the durable benefits of effective therapy. Future approaches should incorporate susceptibility-guided or molecular resistance testing where feasible.^16^

## Supplementary Materials

Supplementary Material

## Figures and Tables

**Table 1.** Baseline Characteristics of the Cohort (n = 2000)

Characteristic	n	%
Age, mean ± SD (years)	2000	77.5 ± 7.8
Male sex	1004	50.2
Female sex	996	49.8
Dyspepsia	502	25.1
Reflux	400	20.0
Abdominal pain	316	15.8
Diarrhea	312	15.6
Nausea	268	13.4
Asymptomatic (screening EGD)	202	10.1
Multimorbidity (≥2 chronic conditions)	514	25.7
Hypertension	418	20.9
Diabetes mellitus	290	14.5
Chronic obstructive pulmonary disease	196	9.8
Chronic kidney disease	84	4.2
Polypharmacy (≥5 drugs)	812	40.6
Prior *H*.* pylori* eradication	562	28.1
Current smoker	610	30.5
Regular alcohol use	298	14.9

EGD, esophagogastroduodenoscopy.

The percentages of symptoms were mutually exclusive (sum of 100%). Comorbidities, lifestyle factors, and treatment history are not mutually exclusive and may exceed 100%.

**Table 2.** Baseline Characteristics Stratified by Age Group (n = 2000)

Characteristic	65-74 years (n = 800)	75-84 years (n = 800)	≥85 years (n = 400)	*P*
Age, mean ± SD (years)	69.5 ± 2.7	79.2 ± 2.8	87.1 ± 1.9	–
Male sex, n (%)	410 (51.2)	390 (48.7)	204 (51.0)	.62
Dyspepsia, n (%)	220 (27.5)	165 (20.6)	77 (19.2)	<.05
Reflux, n (%)	190 (23.7)	130 (16.2)	80 (20.0)	<.05
Abdominal pain, n (%)	140 (17.5)	126 (15.7)	50 (12.5)	.09
Diarrhea, n (%)	130 (16.2)	126 (15.7)	56 (14.0)	.55
Nausea, n (%)	100 (12.5)	115 (14.3)	53 (13.2)	.48
Asymptomatic (screening), n (%)	80 (10.0)	138 (17.2)	84 (21.0)	<.01
Multimorbidity (≥2 chronic conditions), n (%)	170 (21.2)	220 (27.5)	124 (31.0)	<.01
Hypertension, n (%)	160 (20.0)	178 (22.2)	80 (20.0)	.41
Diabetes mellitus, n (%)	118 (14.7)	120 (15.0)	52 (13.0)	.78
Chronic obstructive pulmonary disease, n (%)	65 (8.1)	84 (10.5)	47 (11.7)	.11
Chronic kidney disease, n (%)	28 (3.5)	38 (4.7)	18 (4.5)	.52
Polypharmacy (≥5 drugs), n (%)	280 (35.0)	336 (42.0)	196 (49.0)	<.001
Prior *H*.* pylori* eradication, n (%)	200 (25.0)	236 (29.5)	126 (31.5)	.03
Current smoker, n (%)	270 (33.7)	240 (30.0)	100 (25.0)	.02
Regular alcohol use, n (%)	145 (18.1)	108 (13.5)	45 (11.2)	.01

The percentages of symptoms were mutually exclusive (sum of 100%). Comorbidities, lifestyle factors, and treatment history are not mutually exclusive and may exceed 100%. *P* values were calculated using *χ*^2^ tests or ANOVA where appropriate.

**Table 3.** *H*.* pylori* Prevalence by Age Group (n = 2000)

Age Group	n	Positive, n	Positive, %	*P*-trend
65-74 years	800	466	58.2	
75-84 years	800	448	56.0	
≥85 years	400	216	53.9	*P*-trend = .04
Total	2000	1126	56.3	

The *P*-value was calculated using the Cochran–Armitage test for trends across the age categories.

**Table 4.** *H*.* pylori* Prevalence by Subgroup (n = 2000)

Subgroup	n	Positive, n	Positive, %	*P*
Overall	2000	1126	56.3	
Male	1004	582	57.9	>.05
Female	996	544	54.6	
Prior eradication: Yes	562	176	31.3	<.001
Prior eradication: No	1438	950	66.1	
Current smoker	610	314	51.5	>.05
Non-smoker	1390	812	58.4	
Alcohol user	298	158	53.0	>.05
Non-user	1702	968	56.9	
Polypharmacy (≥5 drugs): Yes	812	480	59.1	>.05
Polypharmacy (≥5 drugs): No	1188	646	54.4	

*P* values represent comparisons within binary subgroups (e.g., male vs. female, smoker vs. non-smoker). For categories in which no direct comparison was applied (e.g., overall prevalence), the cell was left blank.

**Table 5.** Endoscopic Findings and *H*.* pylori P*ositivity (n = 2000)

Endoscopic Diagnosis	n	% of Cohort	*H*.* pylori* Positive, n (%)
Pangastritis	448	22.4	260 (58.0)
Antral gastritis	402	20.1	226 (56.2)
Corpus gastritis	194	9.7	108 (55.7)
Erythematous gastritis	154	7.7	82 (53.2)
Atrophic gastritis	194	9.7	118 (60.8)
Reflux esophagitis	146	7.3	58 (39.7)
Gastric ulcer	120	6.0	54 (45.0)
Duodenal ulcer	88	4.4	48 (54.5)
Gastric polyps	102	5.1	40 (39.2)
Duodenitis	86	4.3	44 (51.2)
Normal endoscopy	66	3.3	28 (42.4)

Patients could have had more than 1 endoscopic finding. Percentages reflect the frequency of each diagnosis within the cohort and do not necessarily sum up to 100%.

**Table 6.** Endoscopic Findings Stratified by Age Group (n = 2000)

Endoscopic Diagnosis	65-74 Years (n = 800)	75-84 Years (n = 800)	≥85 Years (n = 400)	*P*-trend
Pangastritis, n (%)	152 (19.0)	190 (23.7)	106 (26.5)	<.01
Antral gastritis, n (%)	182 (22.7)	160 (20.0)	60 (15.0)	.03
Corpus gastritis, n (%)	70 (8.7)	85 (10.6)	39 (9.7)	.41
Erythematous gastritis, n (%)	72 (9.0)	62 (7.7)	20 (5.0)	.04
Atrophic gastritis, n (%)	52 (6.5)	92 (11.5)	50 (12.5)	<.001
Reflux esophagitis, n (%)	84 (10.5)	46 (5.7)	16 (4.0)	<.001
Gastric ulcer, n (%)	52 (6.5)	46 (5.7)	22 (5.5)	.28
Duodenal ulcer, n (%)	34 (4.2)	38 (4.7)	16 (4.0)	.77
Gastric polyps, n (%)	44 (5.5)	40 (5.0)	18 (4.5)	.63
Duodenitis, n (%)	32 (4.0)	38 (4.7)	16 (4.0)	.81
Normal endoscopy, n (%)	36 (4.5)	43 (5.4)	20 (5.0)	.62

Patients could have had more than 1 endoscopic finding. Percentages reflect the frequency of each diagnosis within each age group and do not necessarily sum up to 100%. The *P* value was calculated using the Cochran–Armitage test for trends across the age categories.

## Data Availability

The data that support the findings of this study are available on request from the corresponding author.
