# Supplementary material for: Prevalence of Helicobacter pylori Infection in Geriatric Adults: A Single-Center Cohort Study from Türkiye
Source: Turk J Gastroenterol. 2026 Jan 5;37(3):380–6. doi: 10.5152/tjg.2025.25499 (PMC12994429; doi:10.5152/tjg.2025.25499)
Supplement: Supplementary Material [file supplementary_material.pdf]

Supplementary Table 1. Histopathologic findings and H. pylori positivity (n = 2000)

Histopathologic feature	n	% of cohort	H. pylori positive, n (%)
Chronic inflammation	706	35.3	403 (57.0%)
Atrophic gastritis	410	20.5	248 (60.5%)
Intestinal metaplasia	384	19.2	192 (50.0%)
Dysplasia (suspected)	84	4.2	34 (40.5%)

Histopathological features were defined according to the updated Sydney classification system. Patients could have had more than one histopathological finding. Percentages reflect the frequency of each finding within the cohort and do not necessarily sum up to 100%.

Supplementary Table 2. Histopathologic findings stratified by age group (n = 2000)

Histopathologic feature	65–74 years (n=800)	75–84 years (n=800)	≥85 years (n=400)	p-trend
Chronic inflammation, n (%)	312 (39.0%)	282 (35.2%)	112 (28.0%)	<0.01
Atrophic gastritis, n (%)	132 (16.5%)	178 (22.2%)	100 (25.0%)	<0.001
Intestinal metaplasia, n (%)	128 (16.0%)	164 (20.5%)	92 (23.0%)	<0.001
Dysplasia (suspected), n (%)	32 (4.0%)	34 (4.2%)	18 (4.5%)	0.77

Histopathologic features were defined according to the updated Sydney classification system. Patients could have had more than one histopathological finding. Percentages reflect the frequency of each finding within each age group and do not necessarily sum up to 100%. The p-value was calculated using the Cochran–Armitage test for trends across the age categories.
